# A bibliometric analysis of research on the anti-obesity effects of curcumin from 2006 to 2025: knowledge structure, research hotspots, and evolution of frontiers

**DOI:** 10.3389/fnut.2026.1825692

**Published:** 2026-05-28

**Authors:** Shan Liu, Zixian Gao, Jinghao Wang, Yujie Fan, Xiaoming He, Qing Guo

**Affiliations:** 1 School of Humanities and Management, Zhejiang Chinese Medical University, Hangzhou, Zhejiang, China; 2 School of Public Health, Zhejiang Chinese Medical University, Hangzhou, Zhejiang, China; 3 The Second Affiliated Hospital of Zhejiang Chinese Medical University, Hangzhou, Zhejiang, China

**Keywords:** bibliometric analysis, curcumin, knowledge structure, metabolic syndrome, obesity, research hotspots

## Abstract

**Objective:**

To systematically map the development trends, knowledge structure, and evolutionary characteristics of research on the anti-obesity effects of curcumin using bibliometric methods, and to identify current hotspots and future directions.

**Methods:**

Publications on curcumin and obesity published between 2006 and 2025 were retrieved from the Web of Science Core Collection (WoSCC) and Scopus. WoSCC was used as the primary dataset for full bibliometric mapping, while Scopus served as an external validation dataset for trend and keyword-structure consistency. After screening, 709 WoSCC records and 234 Scopus records were included. Bibliometric and visualization analyses were performed using VOSviewer, CiteSpace, and Bibliometrix/Biblioshiny, covering annual publication trends, country and institutional collaborations, author networks, keyword co-occurrence, co-citation patterns, clustering, and burst detection.

**Results:**

The annual number of publications demonstrated a quadratic polynomial growth trend (R^2^ = 0.9995), indicating a sustained and accelerating increase in this field. National contribution analysis showed that China ranked first in terms of publication output (167 publications), while the United States occupied a central hub position within international collaboration networks. Keyword co-occurrence and co-citation analyses revealed a distinct evolutionary trajectory of research themes. Early studies primarily focused on fundamental biological mechanisms, such as the anti-inflammatory and antioxidant effects of curcumin. Subsequently, research expanded toward experimental and clinical investigations of obesity-related metabolic disorders, including type 2 diabetes mellitus and non-alcoholic fatty liver disease. In recent years, research frontiers have increasingly shifted toward clinical translation, with particular emphasis on optimizing delivery systems to overcome the limitations of curcumin’s low bioavailability, especially through the development of nano-based formulations. Cross-database validation showed that Scopus and WoSCC exhibited broadly consistent publication trends and keyword structures despite differences in coverage and indexing.

**Conclusion:**

Research on the anti-obesity effects of curcumin has exhibited robust growth, with a clear transition from mechanistic exploration to translational and application-oriented studies. Future efforts should prioritize enhanced international and interdisciplinary collaboration and focus on high-quality clinical studies based on standardized formulations and advanced delivery systems to address bioavailability challenges and facilitate the effective translation of basic research findings into clinical interventions with greater reproducibility and translational relevance.

## 1 Introduction

Obesity has become one of the most pressing global public health challenges. According to data from the World Health Organization (WHO), more than 650 million individuals worldwide are affected by obesity, and its prevalence continues to rise steadily. Obesity substantially increases the risk of serious obesity-related comorbidities, including diabetes mellitus, cardiovascular diseases, and multiple types of cancer (1). Current mainstream strategies for obesity management primarily include lifestyle modification, pharmacological therapy, and metabolic surgery; however, each approach has important limitations. Lifestyle interventions, such as dietary control and physical activity, may achieve short-term weight reduction; however long-term adherence is often poor (2). Pharmacological treatments, including glucagon-like peptide-1(GLP-1) receptor agonists such as semaglutide and lipase inhibitors such as orlistat, have demonstrated efficacy in weight management; however they may be associated with gastrointestinal adverse effects and other safety concerns (3, 4). Although metabolic surgery is highly effective in inducing weight loss, its widespread application is limited by high costs, strict eligibility criteria, and the risk of postoperative complications (5). Consequently, there is an urgent clinical and scientific need to identify safe, effective, and accessible alternative or adjunctive strategies, particularly through the investigation of natural product-derived bioactive compounds.

Curcumin, a polyphenolic compound derived from *Curcuma longa* Linn., is one of the principal active constituents of turmeric and has attracted considerable attention as a bioactive dietary supplement. Owing to its broad biological activities in various human diseases and pathological conditions, curcumin has been recognized as a promising natural candidate for the prevention and management of chronic disorders (6). In recent years, an increasing number of studies have focused on the potential role of curcumin in body weight regulation and the amelioration of obesity-related metabolic abnormalities. Extensive preclinical evidence and preliminary clinical trials suggest that curcumin may exert anti-obesity effects through multiple mechanisms (7, 8), including the suppression of adipose tissue inflammation, the modulation of adipokine secretion, the promotion of lipolysis and the inhibition of adipogenesis, the enhancement of insulin sensitivity, and the regulation of gut microbiota composition (9). Clinical studies have further investigated whether these mechanisms translate into improvements in body weight, body fat distribution, and obesity-associated metabolic disturbances; however, the findings remain heterogeneous and are likely influenced by differences in curcumin formulation, bioavailability, intervention duration, dosage, and study populations (8, 10). With the continuous growth research output in this area, curcumin and obesity research has evolved into an active and multidisciplinary field spanning nutrition science, pharmacology, and endocrinology.

Against this background, the rapid expansion and interdisciplinary nature of the literature necessitate systematic mapping of the developmental trajectory of this research domain. Identifying core contributors and elucidating thematic evolution are essential for synthesizing existing knowledge and guiding future investigations. Bibliometric analysis enables the quantitative assessment of authorship, institutional contributions, keyword distributions, and citation networks, thereby providing objective insights into research hotspots, knowledge structures, and patterns of scientific collaboration (11). In recent years, visualization tools such as VOSviewer and CiteSpace have been widely applied across diverse biomedical fields, offering robust methodological support for the systematic evaluation of research frontiers. However, to date, no comprehensive bibliometric analysis has specifically focused on the anti-obesity effects of curcumin. Consequently, the overall knowledge landscape, dynamic evolution, and collaborative networks in this field remains incompletely characterized. Therefore, the present study aimed to use bibliometric methods to comprehensively analyze research on curcumin and obesity, systematically examining by systematically examining publication trends, core research contributors, thematic hotspots, emerging frontiers, and international collaboration patterns. By doing so, this study seeks to address an important methodological gap, provide an integrated overview of the field, and offer evidence-based insights for future research prioritization and translational development.

## 2 Materials and methods

### 2.1 Data sources and study design

#### 2.1.1 Search strategy

To ensure comprehensiveness and accuracy in literature retrieval, this study analyzed publications from the Web of Science Core Collection (WoSCC) and Scopus, focusing on research related to curcumin and obesity. WoSCC was used as the primary dataset for bibliometric mapping, whereas Scopus was used as an external validation source for publication trends and keyword-based thematic structures. WoSCC was prioritized because it is widely used in bibliometric studies and provides well-curated, standardized bibliographic and cited-reference metadata suitable for co-citation, clustering, and burst-detection analyses. For a multidisciplinary topic such as curcumin and obesity, which spans nutrition, metabolism, pharmacology, and natural products research, WoSCC offers a reliable foundation for identifying knowledge structures, influential publications, and thematic evolution. Although Scopus offers broad coverage, directly merging records from the two databases may introduce heterogeneity due to differences in indexing policies, metadata structures, and update cycles. Therefore, this study adopted a primary-analysis-plus-validation strategy rather than a fully integrated multi-database analysis. For WoSCC, a topic search (TS) was performed by combining keywords related to curcumin and obesity. The detailed search query was as follows: (TS = (*Curcuma longa* OR turmeric OR curcumin OR diferuloylmethane) AND TS = (“overweight” OR “obesit” OR “obese” OR “adiposity” OR “high body mass index” OR “high BMI”)). For Scopus, the search strategy was: TITLE-ABS-KEY ((*Curcuma longa* OR turmeric OR curcumin OR diferuloylmethane) AND (overweight OR obesit OR obese OR adiposity OR “high body mass index” OR “high BMI”)). The publication period was set to 2006–2025, with the language restricted to English and document types limited to Article and Review in both databases to improve comparability between databases and ensure consistency in bibliometric processing. WoSCC was used as the primary database, and retrieval and data export were completed on December 9, 2025, covering the period from January 1, 2006, to December 9, 2025. Scopus was used as a validation database, and supplementary retrieval was conducted on March 4, 2026. Because Scopus time filters are typically year-based, the search window was defined as 2006–2025.

Given the differences in retrieval dates and database update mechanisms, cross-database validation focused on the consistency of annual publication trends and keyword-based thematic patterns, rather than on absolute publication counts. Initial searches identified 855 and 298 potentially relevant records from WoSCC and Scopus, respectively. WoSCC records were exported in TXT and BibTeX formats, whereas full records from Scopus, including citation information, were exported in BibTeX format to ensure data integrity. Because the core analyses were based on WoSCC, subsequent screening and bibliometric procedures were performed primarily on the WoSCC dataset, whereas the Scopus dataset was retained for external validation.

#### 2.1.2 Data processing and bibliometric analysis

**Figure 1:**
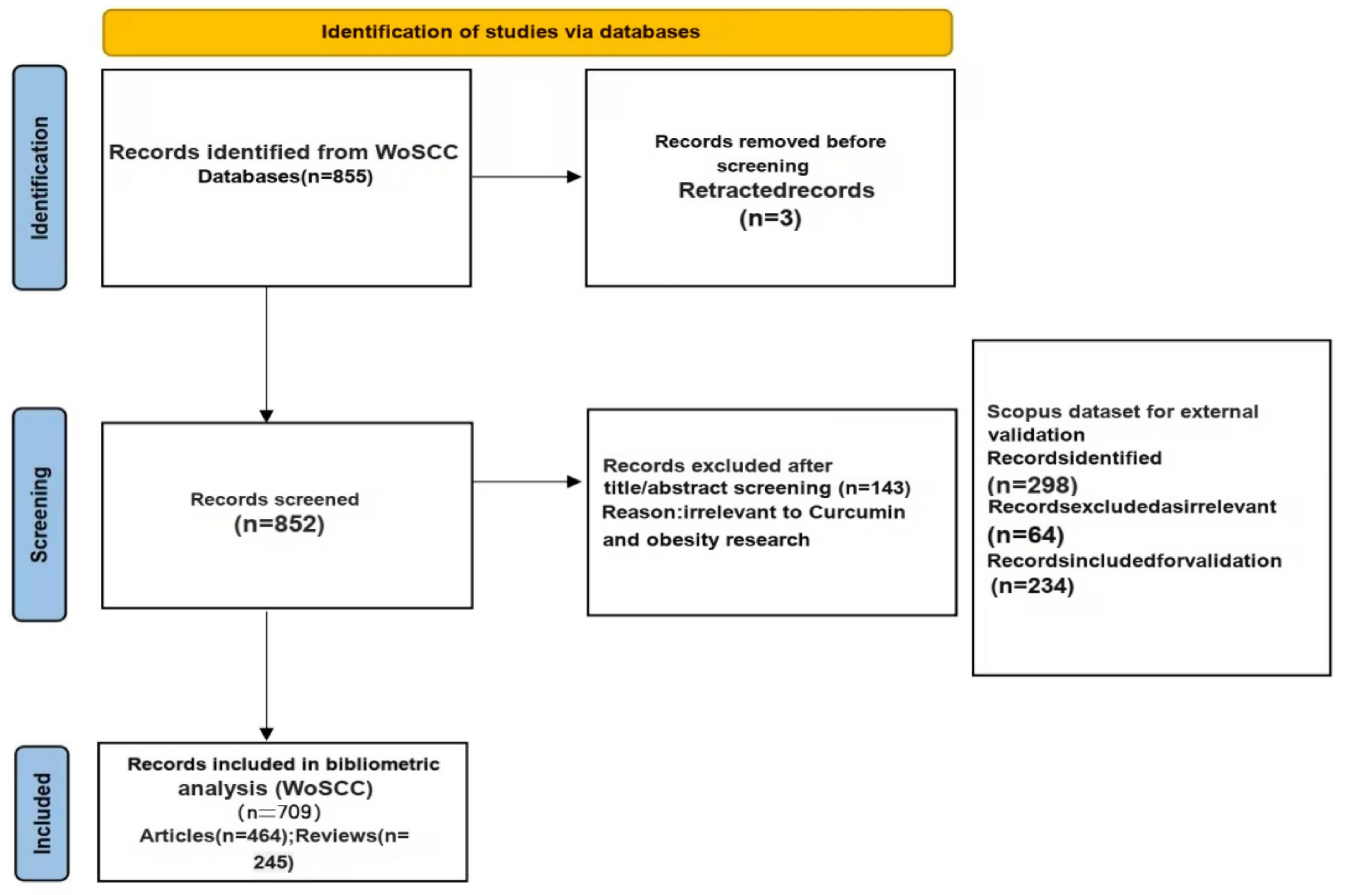
Flowchart of literature retrieval, screening, and inclusion. The Web of Science Core Collection (WoSCC) was used as the primary database for bibliometric analysis. Of the 855 records initially identified, 3 retracted records were removed before screening, and 143 records were excluded after title/abstract review as irrelevant to curcumin and obesity research, resulting in 709 included publications. Scopus was used as an external validation database; of the 298 records retrieved, 234 were retained after exclusion of irrelevant records.

Because WoSCC and Scopus differ in coverage, indexing rules, and metadata fields, this study did not directly merge the two databases. Instead, a separate-database analysis with cross-validation was performed, with WoSCC serving as the primary analytical dataset and Scopus as the validation dataset. To ensure cross-database comparability, record matching and deduplication between WoSCC and Scopus were conducted in RStudio (v2025.09.2) using a Bibliometrix-based workflow. A hierarchical matching rule was applied, with DOI matching performed first and title matching second: records were first matched using normalized DOI strings (with prefix removal, whitespace trimming, and lowercase harmonization), and records without DOI were subsequently matched using normalized titles. Overlapping records, database-specific records, and the final number of unique records after deduplication were summarized in Supplementary Table S1. This matching procedure was conducted to assess overlap and comparability between the two databases, rather than to merge them into a single analytical dataset. To minimize potential bias from automated retrieval, manual screening was combined with data cleaning, and the titles and abstracts of all retrieved WoSCC records were independently reviewed by two researchers. A total of 143 records unrelated to curcumin and obesity were excluded, and 3 retracted papers were further removed. Ultimately, 709 publications met the inclusion criteria and were included in the subsequent analysis, comprising 464 original articles and 245 review articles. After irrelevant entries were removed, 234 valid records were retained from Scopus for external validation. The screening process followed the PRISMA 2020 framework and was illustrated in Figure 1 (12).

Following standardization of bibliographic information and removal of duplicate records, the finalized dataset was exported to multiple analytical tools, including VOSviewer (13), CiteSpace (14, 15), the Bibliometrix R package, Microsoft Office Excel, and Scimago Graphica (16, 17). The bibliometric analysis covered annual publication trends, country and regional distribution, institutional contributions, author collaboration networks, journals, highly cited publications, and keyword co-occurrence and evolution. These analyses were conducted to characterize the knowledge structure, research hotspots, and developmental trajectory of curcumin research in the context of obesity.

## 3 Results

Using the WoSCC dataset, a bibliometric analysis of publications related to curcumin and obesity was conducted using the Bibliometrix package implemented in RStudio. A total of 709 publications published between 2006 and 2025 were ultimately included in the analysis. Overall, this research field showed a pronounced growth trend over the past two decades, with an average annual publication growth rate of 19.53%, indicating that it is a rapidly expanding area of research. The mean citation count per publication was 49.70, reflecting a relatively high overall academic impact. In terms of scientific collaboration, a total of 4,462 authors contributed to the included publications, with an average of 7.87 authors per article. The proportion of internationally co-authored publications was 27.79%, suggesting a high level of collaboration and a growing degree of internationalization within this research domain (Supplementary Table S2).

### 3.1 Annual publication output and growth trends

**Figure 2:**
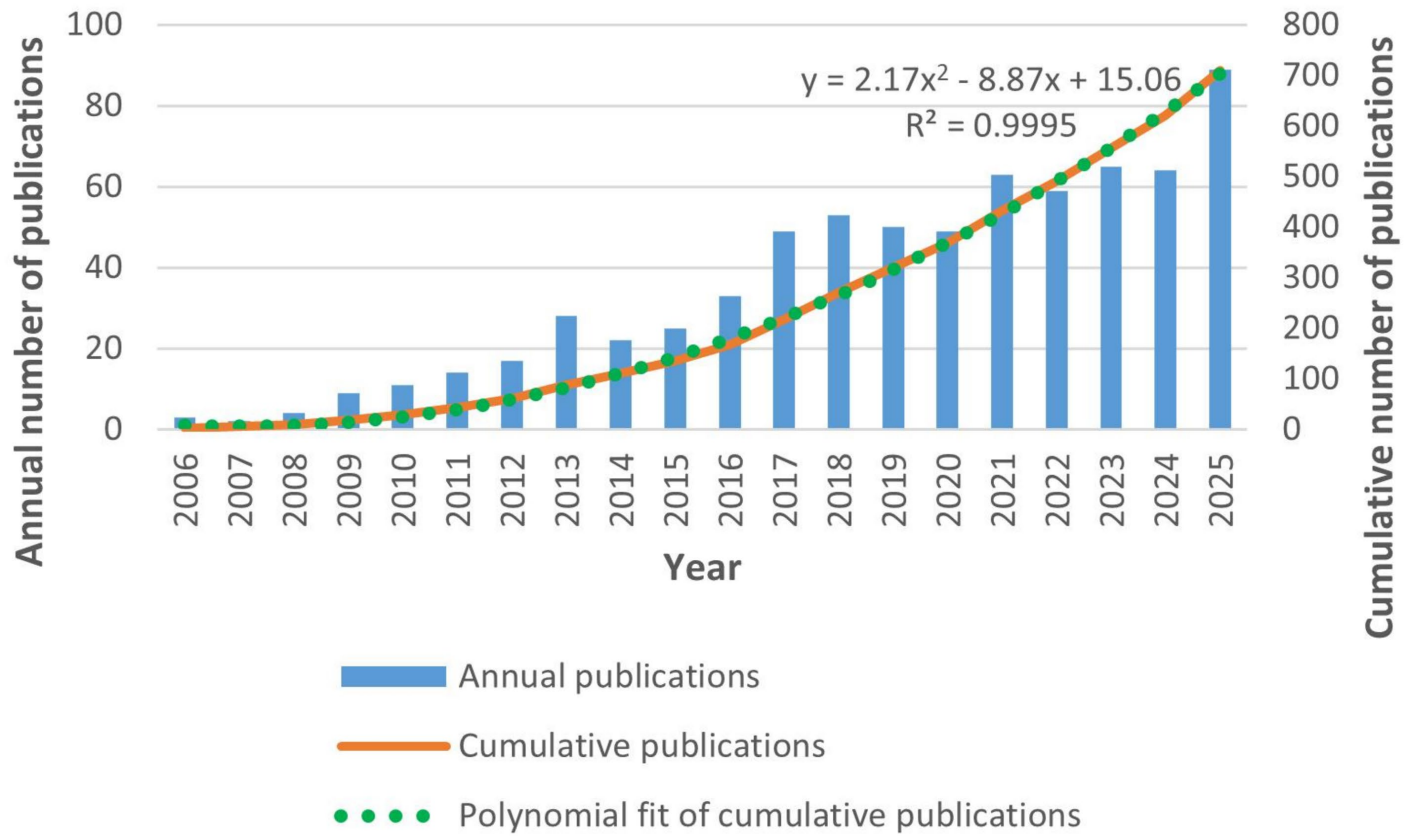
Annual and cumulative publication trends in curcumin and obesity research from 2006 to 2025. Blue bars represent annual publications, the orange line represents cumulative publications, and the green dotted line indicates the polynomial fit of cumulative publications.

The annual and cumulative numbers of publications related to the anti-obesity effects of curcumin from 2006 to 2025 are presented in Figure 2. The annual number of publications is shown as blue bars, whereas the cumulative publication output is represented by an orange curve. Overall, publication output increased steadily over time, although the growth rate varied across periods. A parallel analysis based on Scopus data within the same time window (2006–2025) is presented in Supplementary Figure S3. Although WoSCC and Scopus differed in coverage, the year-by-year trajectory in Scopus was largely consistent with that in WoSCC, with both showing a sustained upward trend overall. Both databases also showed high publication activity in recent years, particularly after 2020, indicating continued growth in interest in curcumin–obesity research. These findings support the reliability of the WoSCC-based primary trend analysis.

Specifically, during 2006 to 2013, the annual number of publications remained relatively low, with fewer than 20 articles published per year on average, indicating that research activity was limited in the early stage of this field. From 2014 onward, annual publication output began to increase gradually, and a more pronounced growth phase was observed after 2017, with the annual number of publications exceeding 50. By 2023, more than 60 articles were published annually, demonstrating sustained and increasing research interest in this topic. Regarding cumulative publication output, a monotonically increasing trend was observed throughout the study period. Notably, the rate of cumulative growth accelerated after 2020. To quantitatively assess the trend in cumulative publications, a quadratic polynomial model was fitted, expressed as: y = 2.17x^2^–8.87x + 15.06, where x represents the sequential year index since 2006. The coefficient of determination (R^2^) for the model was 0.9995, indicating excellent goodness of fit. These results suggest that the polynomial model effectively captured the growth pattern of cumulative publications, and the trend line indicates that research output in this field is expected to continue increasing in the coming years.

### 3.2 Countries, authors, and research institutions

#### 3.2.1 National research output and international collaboration patterns

**Figure 3:**
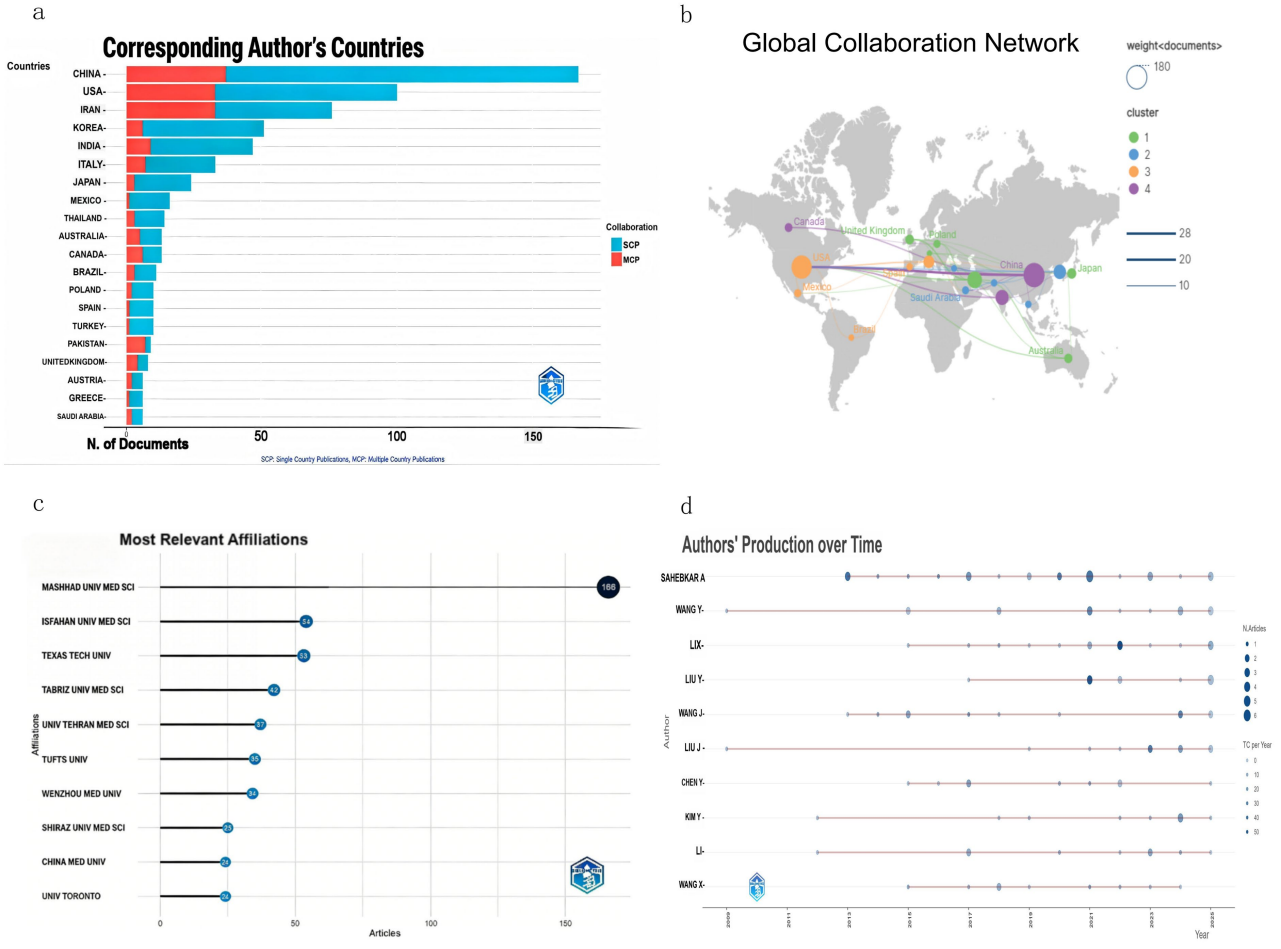
Bibliometric analysis of countries, institutions, and authors in curcumin and obesity research. **(a)** Total publication output by country, with bars showing single-country publications (SCP) and multiple-country publications (MCP). **(b)** International collaboration network among countries; node size is proportional to the number of publications, colors indicate clusters, and connecting lines represent collaboration strength between countries. **(c)** Top 10 institutions by publication volume. **(d)** Publication trajectories of the top 10 most productive authors over time, with bubble size representing annual publication output.

Marked disparities were observed among countries in publication output and collaboration patterns in curcumin and obesity research, as reflected by total publications, single-country publications (SCP), and multiple-country publications (MCP) (Figure 3a, Table 1). China ranked first in total publication output with 167 publications, accounting for 23.60% of all included records. The United States ranked second with 100 publications (14.10%), followed by Iran with 76 publications (10.70%). Other major contributors included South Korea (51 publications), India (47 publications), Italy (33 publications), Japan (24 publications), Mexico (16 publications), Thailand (14 publications), Australia (13 publications), and Canada (13 publications).

**Table 1:** Countries contributing more than 10 publications.

Country	Articles	Articles %	SCP	MCP	MCP %
CHINA	167	23.55	130	37	22.16
USA	100	14.10	67	33	33.00
IRAN	76	10.72	43	33	43.42
KOREA	51	7.19	45	6	11.76
INDIA	47	6.63	38	9	19.15
ITALY	33	4.65	26	7	21.21
JAPAN	24	3.39	21	3	12.50
MEXICO	16	2.26	15	1	6.25
THAILAND	14	1.97	11	3	21.43
AUSTRALIA	13	1.83	8	5	38.46
CANADA	13	1.83	7	6	46.15
BRAZIL	11	1.55	8	3	27.27
POLAND	10	1.41	8	2	20.00
SPAIN	10	1.41	9	1	10.00
TURKEY	10	1.41	9	1	10.00

SCP, single-country publications; MCP, multiple-country publications.

International collaboration also varied considerably across countries. Canada, Iran, and Australia showed relatively high proportions of MCPs, with MCP rates of 46.15, 43.42, and 38.46%, respectively, indicating strong engagement in international collaboration. In contrast, publications from China and South Korea were predominantly single-country publications, with comparatively higher SCP proportions, suggesting that research output from these countries was more often generated through domestic collaboration (Table 1).

#### 3.2.2 Country collaboration network analysis

The country-level collaboration network is presented in Figure 3b. A total of 19 countries were identified in the global collaboration network, which was grouped into four major clusters, reflecting distinct patterns of international cooperation. Cluster 1 (green) primarily included Iran, the United Kingdom, Japan, Australia, Poland, and Austria. Iran occupied a central position within this cluster and showed the highest publication output and citation frequency among its members. Cluster 2 (blue) was composed mainly of Asian countries, including South Korea, Saudi Arabia, Pakistan, Thailand, and Turkey, with South Korea showing relatively high publication output and academic impact within this group. Cluster 3 (orange) included the United States, Italy, Mexico, Spain, and Brazil. Within this cluster, the United States was the most prominent contributor, ranking first among all countries in terms of the number of collaboration links (19), total link strength (120), and total citations (14,193). Cluster 4 (purple) included China, India, and Canada, forming a group characterized by relatively dense intercontinental collaboration.

#### 3.2.3 Distribution of major research institutions

The distribution of major contributing institutions in curcumin and obesity research is shown in Figure 3c. Among the 1,236 institutions identified, the top 10 institutions by publication volume were MASHHAD UNIV MED SCI (166 publications), ISFAHAN UNIV MED SCI (54), TEXAS TECH UNIV (53), TABRIZ UNIV MED SCI (42), UNIV TEHRAN MED SCI (37), TUFTS UNIV (35), WENZHOU MED UNIV (34), SHIRAZ UNIV MED SCI (25), CHINA MED UNIV (24), and UNIV TORONTO (24). Notably, Iranian institutions accounted for five of the top ten most productive institutions. Mashhad University of Medical Sciences (MASHHAD UNIV MED SCI) exhibited a markedly higher publication output than all other institutions, highlighting its leading position in this research field. In terms of institutional types, medical universities and medical research centers represented the predominant research entities, although comprehensive universities and other research institutions were also involved. Overall, this pattern underscores the multidisciplinary nature of research on curcumin and obesity.

#### 3.2.4 Analysis of core authors

Author-level analysis showed that six authors published 10 or more papers during the study period. Among them, SAHEBKAR A was the most productive author with 28 publications, followed by WANG Y (16 publications) and LI X (15 publications). As shown in Figure 3d, SAHEBKAR A maintained sustained publication activity over time, indicating continued contributions to this research field.

### 3.3 Analysis of source journals

**Figure 4:**
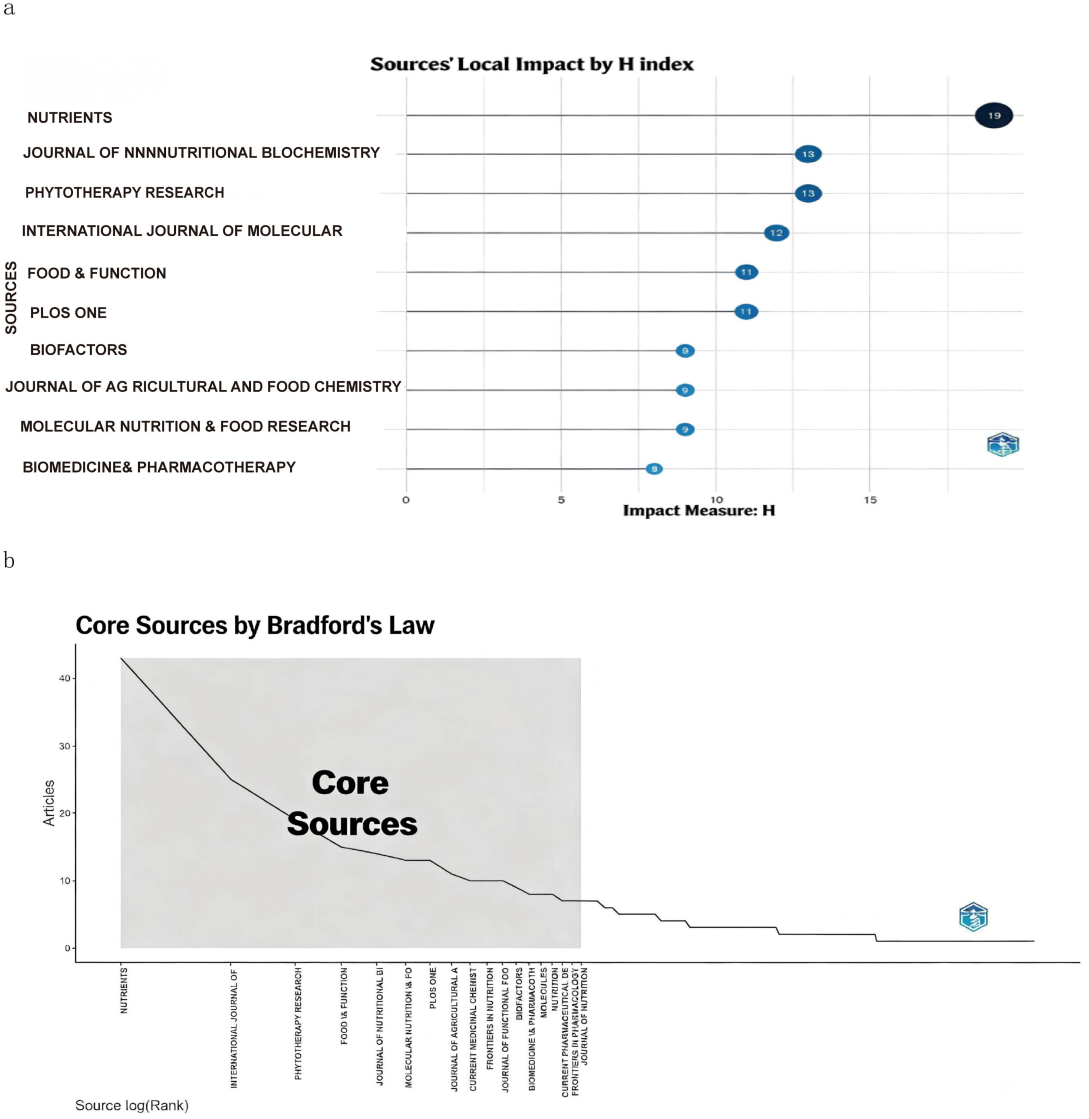
Analysis of source journals in curcumin and obesity research: **(a)** Local impact of journals with at least 10 publications, measured by the *H*-index and **(b)** Core source distribution based on Bradford’s law. The vertical axis shows the number of publications, and the horizontal axis shows source rank on a logarithmic scale [Source log(Rank)]. The shaded region denotes the core source zone, representing the journals that contributed the largest share of publications in this field.

To identify the major publication venues in the field of curcumin and obesity research, a bibliometric analysis of journal productivity and local influence was conducted. Figure 4a presents the leading journals with at least 10 publications and their corresponding H-indices. Among these journals, Nutrients ranked first with 43 publications and an *H*-index of 19, followed by the International Journal of Molecular Sciences with 25 publications (*H*-index = 12). Phytotherapy Research (19 publications, *H*-index = 13), Food and Function (15 publications, *H*-index = 11), and the Journal of Nutritional Biochemistry (14 publications, *H*-index = 13) ranked next among the leading journals.

Additional citation analysis revealed substantial variation in journal-level influence. Publications in Nutrients accumulated a total of 1,682 citations, indicating substantial overall visibility within the field. In contrast, although the Journal of Nutritional Biochemistry published a relatively smaller number of papers (14 publications), it achieved a total of 1,926 citations, ranking first among the included journals in terms of total citation count. This finding suggests that publications in this journal had a relatively high academic impact in this research domain.

Journal source distribution was further analyzed based on Bradford’s Law, as illustrated in Figure 4b. The core source zone was composed of a small number of highly productive journals, including Nutrients, the International Journal of Molecular Sciences, Phytotherapy Research, and Food and Function, which collectively accounted for a disproportionately large share of publications. As journal rank increased, the number of publications declined sharply, with the majority of journals contributing only a limited number of papers. This distribution pattern indicates that research outputs in the field of curcumin and obesity are highly concentrated in a small group of core journals.

### 3.4 Keyword analysis

Keyword analysis was performed to identify major research themes the knowledge structure and temporal trends in curcumin and obesity research. In this study a comprehensive analysis of research topics in the field of curcumin and obesity was conducted by integrating keyword word cloud visualization high-frequency keyword co-occurrence network analysis clustering analysis and burst detection

#### 3.4.1 High-frequency keywords and word cloud analysis

**Figure 5:**
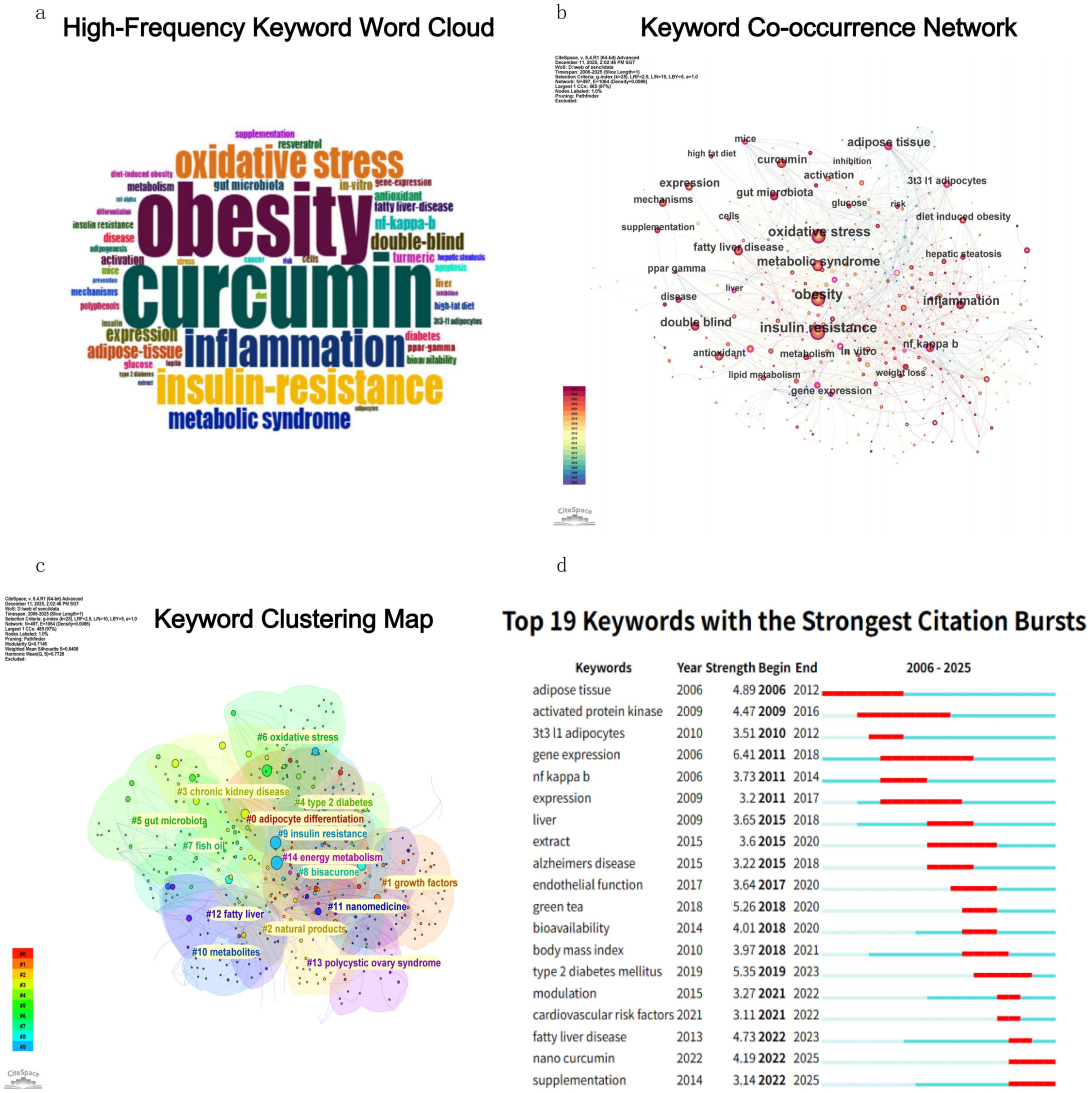
Keyword analysis of curcumin and obesity research. **(a)** Word cloud of high-frequency keywords, with larger fonts indicating higher occurrence frequency. **(b)** Keyword co-occurrence network showing relationships among major research topics; node size reflects keyword frequency, and link thickness indicates co-occurrence strength. **(c)** Keyword clustering map generated by CiteSpace, in which different colors represent distinct thematic clusters and cluster labels indicate the main research topics. **(d)** Top 19 keywords with the strongest citation bursts, illustrating the temporal evolution of research hotspots from 2006 to 2025.

Keyword word cloud analysis was employed to visually represent the distribution of high-frequency terms with font size proportional to their frequency of occurrence across all included publications. As shown in Figure 5a “curcumin” (358 occurrences) and “obesity” (351 occurrences) were the two most frequently appearing keywords constituting the central research focus of this field. In addition to these core terms keywords such as “inflammation” (182 occurrences) “insulin resistance” (172 occurrences) “oxidative stress” (159 occurrences) and “metabolic syndrome” (98 occurrences) also appeared with high frequency. These terms reflect sustained research attention to inflammatory responses metabolic dysregulation and oxidative stress in studies investigating curcumin and obesity. Overall the high-frequency keywords encompassed multiple dimensions of the research field including disease phenotypes and metabolic outcomes molecular and signaling mechanisms as well as experimental models and methodological approaches.

#### 3.4.2 Keyword co-occurrence network analysis

Keyword co-occurrence network analysis was conducted to elucidate the relational structure among different research themes by mapping co-occurrence relationships between keywords. In this network nodes represent keywords with node size indicating keyword frequency while edges denote co-occurrences within the same publications and edge thickness reflects co-occurrence strength.

As illustrated in Figure 5b, the co-occurrence network exhibited a highly connected structure centered on “curcumin” and “obesity” The node “curcumin” was positioned at the core of the network and showed strong co-occurrence relationships with keywords such as “oxidative stress,” “insulin resistance,” “inflammation,” and “adipose tissue.” In parallel, the keyword “obesity” was strongly associated with disease-related terms, including “metabolic syndrome,” “type 2 diabetes,” and “fatty liver disease.” Further analysis of betweenness centrality showed that although “curcumin” and “obesity” had centrality values of 0.07 and 0.02, respectively, these were not the highest within the network. Keywords such as “adipose tissue” (0.14), “activated protein kinase” (0.11), and “NF-kappa B” (0.10) exhibited higher centrality values, indicating that these terms functioned as key connectors linking multiple research themes.

#### 3.4.3 Keyword clustering analysis

Keyword clustering analysis was performed to identify relatively stable thematic groups and characterize their internal structures. As shown in Figure 5c a total of 15 keyword clusters were identified forming a multicenter and interconnected network. All clusters exhibited silhouette values greater than 0.70 with the maximum silhouette value reaching 0.94 indicating high internal consistency and robust clustering quality. Among the major clusters cluster #9 (“insulin resistance”) showed close connections with cluster #0 (“adipocyte differentiation”) cluster #5 (“gut microbiota”) and cluster #6 (“oxidative stress”). Cluster #12 (“fatty liver”) was spatially adjacent to cluster #4 (“type 2 diabetes”) suggesting thematic proximity between these research topics. In terms of cluster size cluster #0 (size = 56) cluster #1 (size = 41) and cluster #2 (size = 40) represented the largest thematic groups encompassing topics related to adipocyte biology metabolic abnormalities and natural product research.

#### 3.4.4 Keyword burst detection analysis

Keyword burst detection analysis was conducted to identify terms that experienced sharp increases in research attention during specific time periods thereby reflecting the dynamic evolution of research hotspots. As illustrated in Figure 5d a total of 19 keywords with burst strengths greater than 3 were identified with burst periods spanning from 2006 to 2025.

During the early stage (2006–2012), burst keywords primarily included “adipose tissue,” “activated protein kinase,” and “3 T3-L1 adipocytes,” indicating a research focus on adipose tissue and cellular models. In the middle stage (approximately 2011–2020), keywords such as “gene expression” (burst strength = 6.41), “NF-κB,” “liver,” and “endothelial function” emerged, reflecting an expansion of research themes toward molecular mechanisms and organ-level effects. In the most recent stage (from 2018 onward), burst keywords included “type 2 diabetes mellitus,” “fatty liver disease,” “bioavailability,” “nano curcumin,” and “supplementation,” highlighting a shift in research attention toward specific metabolic complications and application-oriented topics.

#### 3.4.5 Scopus-based keyword validation

To enable cross-database validation, we conducted an author-keyword co-occurrence analysis using Scopus. The resulting network clearly identified “curcumin” and “obesity” as the core themes, consistent with the findings derived from the Web of Science dataset. High-frequency keywords (Supplementary Table S4) included curcumin (*n* = 89), obesity (*n* = 69), turmeric (*n* = 36), inflammation (*n* = 32), *Curcuma longa* (*n* = 28), metabolic syndrome (*n* = 13), diabetes (*n* = 12), oxidative stress (*n* = 12), nutraceuticals (*n* = 10), and insulin resistance (*n* = 9). Network topology (Supplementary Figure S5) indicated that “curcumin” was more closely linked to translational and intervention-oriented topics (e.g., turmeric/*Curcuma longa*, bioavailability, and type 2 diabetes), whereas “obesity” was more strongly associated with mechanism-oriented terms (e.g., adipogenesis, adipocytes, gut microbiota, and anti-obesity). Keywords such as inflammation, insulin resistance, and oxidative stress occupied bridging positions between clusters, suggesting their role as cross-topic connectors.

### 3.5 Co-cited reference analysis

Co-cited reference analysis was performed to identify the knowledge base and structural evolution of curcumin and obesity research. To this end, co-citation clustering and reference burst detection were combined to examine the major knowledge foundations of the field and their temporal dynamics.

#### 3.5.1 Co-cited reference clustering analysis

**Figure 6:**
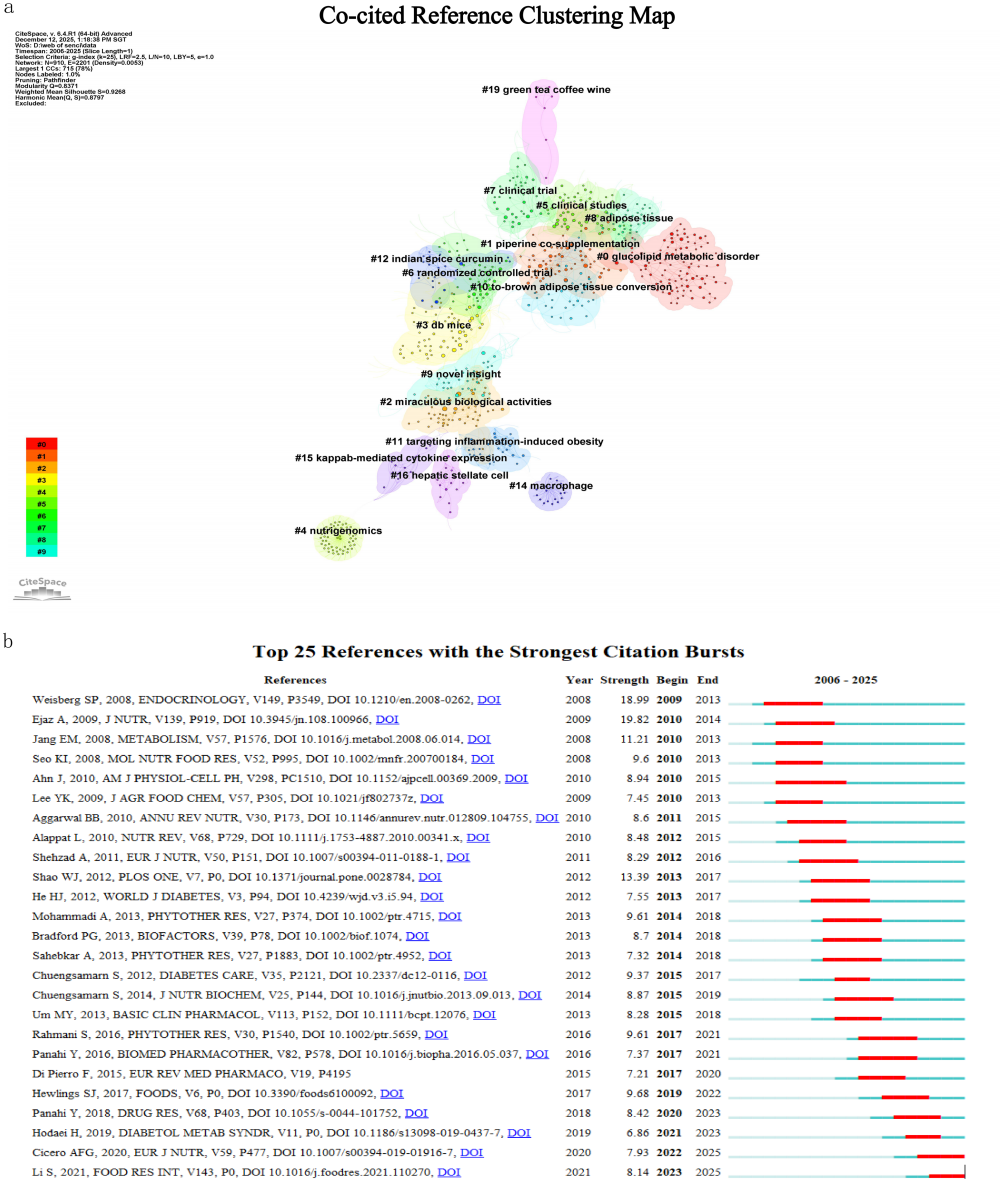
Co-cited reference analysis of curcumin and obesity research. **(a)** Co-cited reference clustering map generated by CiteSpace. Different colors represent distinct thematic clusters, and cluster labels indicate the major knowledge themes in the field. **(b)** Top 25 references with the strongest citation bursts. The red bars indicate the periods during which the references experienced a sharp increase in citations, reflecting shifts in the knowledge base over time.

Co-cited reference clustering analysis was used to identify major thematic groups within the knowledge base. As shown in Figure 6a, the co-citation network of references formed multiple tightly connected clustering modules. A total of 17 co-citation clusters were identified, and all clusters exhibited silhouette values greater than 0.85, indicating high clustering reliability and strong internal consistency. Among all identified clusters, the largest was cluster #0, labeled “disorders of glucose and lipid metabolism,” which contained 103 references and had an average publication year of 2020, suggesting that this topic has occupied a prominent position in recent research. In contrast, smaller clusters were also observed, such as cluster #19 (“green tea, coffee, alcohol”), which included only 7 references. Overall, the co-citation network comprised multiple representative core clusters that together constituted the principal knowledge framework of the field.

From the perspective of thematic composition, clusters #6 (“randomized controlled trial,” mean year = 2013), #7 (“clinical trial,” mean year = 2018), and #5 (“clinical research,” mean year = 2019) mainly encompassed clinical research–related topics and represented important sources of clinical evidence within this field. In contrast, clusters #8 (“adipose tissue,” mean year = 2017), #10 (“brown adipose tissue conversion,” mean year = 2015), and #11 (“targeting inflammation-induced obesity,” mean year = 2006) primarily focused on basic and mechanistic research themes.

Analysis of the temporal distribution of clusters revealed clear temporal patterns in research focus. Early clusters (approximately 2004–2008) were mainly concentrated on inflammatory responses, adipocytes, and associated fundamental mechanisms. Mid-stage clusters (approximately 2012–2017) gradually expanded toward animal models, functional transformation of adipose tissue, and combination intervention strategies. More recent clusters (approximately 2018–2020) increasingly focused on topics closely related to clinical translation, such as gut microbiota and disorders of glucose and lipid metabolism.

#### 3.5.2 Reference burst detection analysis

Reference burst detection analysis was conducted to identify key publications that experienced a significant increase in citation frequency during specific time periods, thereby highlighting critical nodes within the evolving knowledge base. As shown in Figure 6b, a total of 25 references with significant burst characteristics were detected. Among these burst references, the publications with the highest burst strengths included Ejaz a (2009, journal of nutrition, burst strength = 19.82, burst period = 2010–2014) and Weisberg SP (2008, endocrinology, burst strength = 18.99, burst period = 2009–2013), indicating that these studies received concentrated attention during specific periods. In terms of temporal distribution, reference bursts exhibited distinct stage-specific patterns. During the early stage (approximately 2009–2013), burst references were predominantly associated with fundamental mechanistic studies using obesity-related animal models, particularly those focusing on anti-inflammatory and antioxidant effects. In the middle stage (approximately 2013–2018), burst references gradually shifted toward preclinical research on metabolism-related diseases such as diabetes and fatty liver disease. In the most recent stage (approximately 2019–2025), burst references were more frequently related to systematic reviews, safety evaluations of curcumin, and the development of novel delivery systems.

## 4 Discussion

### 4.1 Overview of the field and publication characteristics

Taken together, the bibliometric findings indicate that research on curcumin and obesity represents a continuously expanding and increasingly active domain. Although early-stage attention to this topic was relatively limited, the rapid growth in publication output over the past decade suggests a transition from exploratory work toward more systematic, multi-level investigations. Despite this expansion, substantial room remains for further development, particularly in deepening mechanistic understanding, strengthening clinical validation, and improving translational applicability.

Marked cross-national differences were observed in both research output and collaboration patterns. China ranked first in total publication volume; however, its proportion of multiple-country publications (MCP) was relatively low (22.10%) compared with other high-output countries, such as the United States (33%) and Iran (43%). This pattern suggests that research activities in China are more frequently conducted within domestic teams. In contrast, the United States and Iran not only generated substantial outputs but also demonstrated greater engagement in international collaboration. Some countries, such as Mexico, also exhibited a comparatively low MCP proportion, indicating that the extent of international collaboration remains uneven across regions. Overall, although an international collaboration network has formed in this field, cooperative ties appear to be concentrated among a limited set of core countries, and a highly interconnected, globally distributed collaboration system has not yet been established.

Country-level collaboration network analysis further highlights the United States as a key hub, occupying a central position in cross-national collaboration, network connectivity, and academic influence. Meanwhile, China’s high productivity reflects its substantial investment in research capacity and output scale. The emergence of four major country clusters suggests the presence of regionally coordinated research patterns; however, connectivity between clusters remains relatively limited. Strengthening cross-cluster collaborations—particularly by promoting deeper partnerships between high-output and high-impact countries—may contribute to improving overall research quality and enhancing the international influence of this domain.

At the institutional and author levels, research capacity shows a clear tendency toward concentration. Multiple Iranian medical universities ranked among the most productive institutions, with Mashhad University of Medical Sciences (MASHHAD UNIV MED SCI) leading by a wide margin. This high institutional productivity appears to be closely associated with the sustained contributions of the core author, SAHEBKAR A, who also demonstrated leading performance in publication output and H-index and maintained active international collaborations. Such sustained scholarly productivity likely contributes to Iran’s prominent position in this field and underscores the role of influential researchers in shaping the development of specialized domains. In the field of curcumin and obesity research, the academic impact of SAHEBKAR A was primarily established through a series of highly cited clinical studies published between 2013 and 2020. These studies, which used randomized controlled and crossover trial designs, systematically demonstrated the efficacy of curcumin and curcuminoid formulations in improving obesity-related dyslipidemia, redox balance, and chronic low-grade inflammation (18–20). Subsequently, investigations conducted in populations with metabolic syndrome further elucidated the modulatory effects of curcumin on inflammatory mediators and cardiometabolic risk markers, and these findings became among the most frequently cited clinical evidence in this field (21, 22). Building on this foundation, a double-blind randomized controlled trial published in 2020 extended the research focus to obesity-associated non-alcoholic fatty liver disease, marking a transition from isolated metabolic parameters to the management of multisystem obesity-related complications (23). Studies published after 2020 have increasingly emphasized both mechanistic exploration and broader clinical applicability, although their citation impact is still accumulating.

Journal distribution patterns provide additional insight into the publication ecology and disciplinary orientation of the field. Nutrients performed prominently in both publication volume and H-index, serving as one of the most important outlets for research on curcumin and obesity. Although journals such as the Journal of Nutritional Biochemistry and Phytotherapy Research published fewer relevant articles, their publications accrued high citation counts, suggesting that studies focusing on nutritional biochemistry and plant-derived bioactive compounds exert substantial academic influence in this domain. This observation indicates that research impact is not solely determined by output volume but is also closely associated with research depth and journal scope. Overall, high-impact outlets in this field are largely concentrated in nutrition, functional foods, and phytotherapy, whereas participation by pharmacy and clinical medicine journals remains relatively limited. This disciplinary imbalance further suggests that the field remains driven largely by nutrition- and natural-product-oriented research, whereas clinically oriented dissemination and pharmaceutical standardization studies remain comparatively underrepresented.

In addition, the source distribution conforms to Bradford’s Law, reflecting a “concentration–dispersion” pattern in which a small set of core journals contributes a disproportionate share of publications. This concentration facilitates efficient access to key literature and enables researchers to track emerging trends; however, it may also constrain the diffusion of findings into broader disciplinary communities. Therefore, while consolidating publication presence in core journals, expanding dissemination into journals in clinical medicine, pharmaceutics, and translational medicine may help enhance the interdisciplinary reach and practical impact of research on curcumin and obesity. Such diversification may also help bridge the current gap between mechanistic accumulation and clinical translation.

### 4.2 Research hotspots, knowledge evolution, and translational challenges

#### 4.2.1 Current research hotspots and core mechanisms

Keyword analysis indicates that current research hotspots in the field of curcumin and obesity are predominantly centered on core pathological mechanisms particularly chronic low-grade inflammation and oxidative stress associated with obesity. Keywords such as “NF-κB,” “oxidative stress,” “adipose tissue,” and “insulin resistance” showed prominent positions in co-occurrence networks and high centrality values. Rather than merely indicating topic frequency these patterns suggest that the field is structured around a mechanistic axis linking inflammation redox imbalance adipose tissue dysfunction and insulin resistance. Within this framework curcumin has been widely investigated as a multi-target modulator of interconnected signaling pathways and adipose tissue function with potential relevance to systemic metabolic homeostasis. This hotspot profile is broadly consistent with previous experimental evidence on the anti-inflammatory antioxidant and metabolic regulatory properties of curcumin. At the same time the thematic scope of research has clearly expanded beyond simple regulation of body weight or fat accumulation to encompass systemic metabolic disorders including type 2 diabetes mellitus fatty liver disease and metabolic syndrome. This thematic expansion suggests that research on curcumin has increasingly moved beyond isolated weight-related outcomes toward a broader metabolic disease framework rather than being limited to anti-obesity effects alone. This broader thematic profile also provides a basis for subsequent discussion of how mechanistic findings relate to translational and clinical research.

#### 4.2.2 Evolution of knowledge structure and paradigm shifts

Keyword clustering and temporal evolution analyses further reveal a broadly layered thematic structure within this research field. Broadly the identified research themes may be interpreted as spanning three interrelated levels: (i) fundamental mechanistic studies focusing on adipose tissue signaling pathways and oxidative stress; (ii) disease-oriented research targeting metabolic conditions such as type 2 diabetes and fatty liver disease; and (iii) emerging interdisciplinary frontiers represented by gut microbiota modulation nano-based delivery systems and multi-component synergistic strategies. This interpretation is supported by both keyword burst detection and reference burst analysis: Early bursts were mainly associated with adipose tissue activated protein kinase 3T3-L1 adipocytes and obesity-related animal models; mid-stage bursts involved gene expression

NF-κB liver endothelial function diabetes and fatty liver disease; and recent bursts increasingly included type 2 diabetes mellitus fatty liver disease bioavailability nano-curcumin supplementation systematic reviews safety evaluation and novel delivery systems. Together these patterns suggest a gradual shift in research attention from mechanism-centered exploration toward disease-oriented investigation and more recently toward application-related and translational topics.

It is noteworthy that although emerging topics such as the “gut microbiota–metabolism axis” and “nano-curcumin” have shown a marked increase in attention in recent years, their integration with core mechanism-centered themes appears less prominent in the current bibliometric maps. For example, gut microbiota appeared as a distinct cluster and was connected to insulin resistance, whereas nano-curcumin and bioavailability were mainly identified as recent burst terms rather than as established core nodes in the co-occurrence network. This pattern suggests that some emerging directions are gaining attention rapidly, but their relationships with the established mechanistic framework of inflammation, oxidative stress, adipose tissue remodeling, and insulin resistance are less clearly reflected in the current knowledge structure.

#### 4.2.3 Knowledge base and translational bottlenecks

Findings from co-cited reference clustering and burst detection analyses provide further validation of the evolutionary trajectory described above. Early burst references primarily established the foundational mechanisms through animal models and cellular experiments, demonstrating the capacity of curcumin to ameliorate obesity-related inflammation and metabolic disturbances. Subsequent influential studies increasingly focused on preclinical and early-stage clinical investigations of specific metabolic diseases, such as diabetes and fatty liver disease. More recent burst references have concentrated on systematic reviews, strategies to enhance bioavailability, and the development of novel delivery systems, indicating a paradigm shift toward translational evaluation and application optimization.

Nevertheless, burst analysis also highlights critical limitations within the current knowledge base. The co-citation structure indicates that the knowledge base remains more strongly grounded in mechanistic and preclinical studies than in mature clinical evidence. Although clinical research–related clusters, including “randomized controlled trial,” “clinical trial,” and “clinical research,” have emerged as identifiable components of the field, they do not appear to dominate the knowledge base to the same extent as mechanism-centered and metabolism-related clusters. In addition, the coexistence of clinical evidence clusters and emerging mechanistic clusters within relatively distinct thematic modules suggests that the translation of mechanistic insights into clinically consolidated evidence remains an ongoing process.

### 4.3 Biological mechanisms and implications for clinical translation

**Figure 7:**
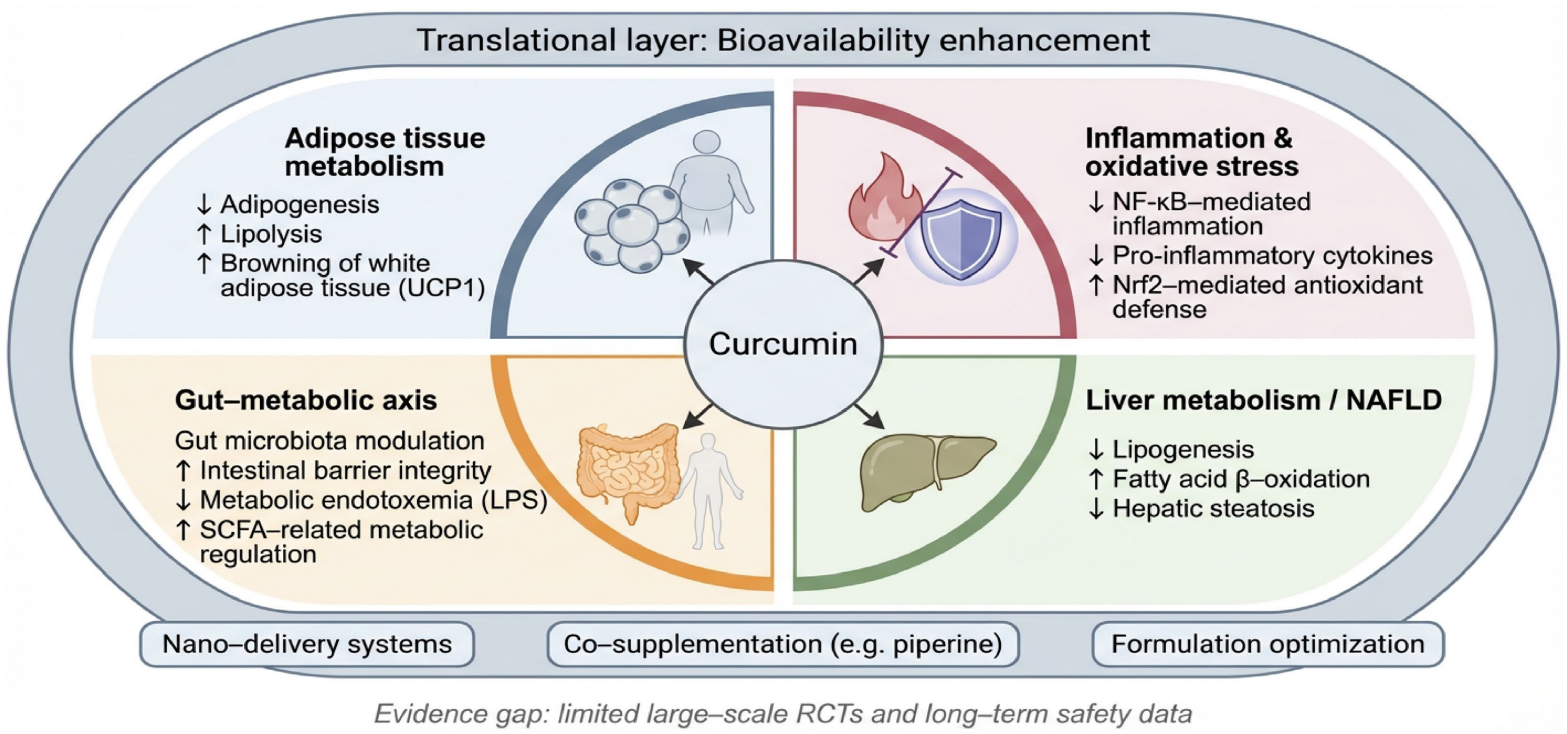
Integrative mechanistic framework underlying the anti-obesity effects and translational potential of curcumin. Curcumin may regulate obesity-related metabolic dysfunction through effects on adipose tissue metabolism, inflammation and oxidative stress, liver metabolism, and the gut-metabolic axis. Strategies such as nano-delivery systems, co-supplementation, and formulation optimization may improve its bioavailability and clinical applicability. UCP1, uncoupling protein 1; NF-κB, nuclear factor kappa B; Nrf2, nuclear factor erythroid 2-related factor 2; NAFLD, non-alcoholic fatty liver disease; LPS, lipopolysaccharide; SCFA, short-chain fatty acid; RCTs, randomized controlled trials.

Bibliometric analysis indicates that research hotspots related to curcumin and obesity are characterized by a highly clustered structure, mainly focusing on adipose tissue regulation, inflammation, oxidative stress, metabolic complications, and formulation optimization (Figure 7). This thematic pattern is consistent with the biological properties of curcumin, which exerts regulatory effects across multiple key metabolic nodes (24, 25)

At the level of adipose tissue, both high-frequency keywords and high-centrality nodes were predominantly associated with adipogenesis, lipolysis, and energy metabolism. These findings are highly consistent with previous mechanistic studies showing that curcumin modulates lipid metabolism through the adenosine monophosphate-activated protein kinase (AMPK)–acetyl-CoA carboxylase (ACC)–sterol regulatory element-binding protein-1c (SREBP-1c)/peroxisome proliferator-activated receptor gamma (PPARγ) signaling axis (26). Experimental evidence suggests that curcumin activates AMPK signaling, thereby suppressing the expression of adipogenic transcription factors and lipogenic enzymes while enhancing fatty acid oxidation. In parallel, a research cluster centered on the “conversion of white adipose tissue to brown-like adipose tissue” has emerged, indicating that the enhancement of energy expenditure has become an important research direction in recent years. This process is closely associated with thermogenic regulatory pathways involving PR domain containing 16 (PRDM16), peroxisome proliferator-activated receptor gamma coactivator 1-alpha (PGC-1*α*), and uncoupling protein 1 (UCP1) (27, 28).

Systemic metabolic dysregulation, inflammation, and oxidative stress consistently occupy structurally central positions within the keyword co-occurrence network. Terms such as “nuclear factor kappa B (NF-κB)” and “oxidative stress” not only exhibit high occurrence frequencies but also show strong connective roles across different thematic clusters. Accumulating evidence indicates that curcumin suppresses NF-κB signaling, thereby reducing the expression of pro-inflammatory cytokines such as tumor necrosis factor-α (TNF-α) and interleukin-6 (IL-6), while simultaneously activating the nuclear factor erythroid 2–related factor 2 (Nrf2)–antioxidant response element (ARE) antioxidant defense system to upregulate factors including heme oxygenase-1 (HO-1) and NAD(P)H quinone dehydrogenase 1 (NQO1). This dual regulatory pattern enables the concurrent attenuation of inflammation and oxidative stress and may contribute to improvements in insulin resistance and overall metabolic homeostasis (29, 30).

From a disease-oriented perspective, the growing prominence of fatty liver–related themes in co-citation clusters and keyword burst analyses suggests a shift in research emphasis from body weight or single metabolic indicators toward specific clinical endpoints. Previous studies have demonstrated that curcumin can activate AMPK/SIRT1 signaling, inhibit SREBP-1c-mediated lipogenesis, promote fatty acid *β*-oxidation, and concurrently attenuate hepatic inflammation and oxidative injury, thereby improving metabolic abnormalities associated with non-alcoholic fatty liver disease. This multi-level regulatory profile supports the interpretation of NAFLD as an important disease-oriented model linking fundamental metabolic research with clinical translation (27, 31, 32).

It is noteworthy that although keywords such as “gut microbiota” and “gut axis” currently exhibit less structural prominence than classical mechanism-related terms within the current network, they have become increasingly prominent in recent clusters and temporal evolution analyses, indicating a rapidly developing research frontier. Emerging evidence suggests that curcumin and its metabolites may indirectly modulate host inflammation and energy metabolism by reshaping gut microbial composition, enhancing intestinal barrier integrity, and reducing metabolic endotoxemia. This mechanistic paradigm may partly explain the systemic metabolic effects of curcumin despite its limited oral bioavailability and may also help explain its broader metabolic actions (28, 33).

At the translational level, insufficient bioavailability remains one of the primary constraints limiting the clinical application of curcumin (34). Keyword burst analysis reveals a recent surge in attention toward “bioavailability,” “nano-curcumin,” and “supplementation,” reflecting a broader paradigm shift from mechanistic elucidation to formulation optimization and clinical feasibility assessment (35). Current research efforts have focused on improving *in vivo* stability and absorption efficiency through nano-delivery systems, absorption enhancers such as piperine, and multi-component synergistic strategies (36). Taken together, these findings suggest that strong mechanistic evidence has not yet been fully matched by equally consistent clinical evidence (37). However, translational progress is also constrained by formulation heterogeneity, the lack of standardized dosing regimens, differences in study populations, and the limited availability of high-quality long-term clinical evidence. These considerations suggest that future studies should prioritize standardized formulations, dose–response evaluation, long-term safety assessment, and well-designed randomized controlled trials in clearly defined obesity-related metabolic populations (38).

### 4.4 Clinical evidence for curcumin intervention in obesity

Bibliometric analyses, particularly keyword burst and co-cited reference burst patterns, indicate that research on curcumin and obesity has progressively shifted from a predominantly mechanistic focus toward clinical translation. However, evidence derived from currently published randomized controlled trials (RCTs) and meta-analyses suggests that this translational trend remains uneven across different clinical endpoints. Overall, clinical evidence supporting the effects of curcumin is relatively concentrated on mechanism-related outcomes such as inflammation, oxidative stress, and metabolic regulation, whereas findings regarding traditional obesity endpoints, including body weight and adiposity, remain heterogeneous.

With respect to chronic low-grade inflammation, a core pathological mechanism of obesity, clinical findings show a high degree of concordance with bibliometric keyword co-occurrence analyses highlighting themes such as “NF-κB” and “inflammatory cytokines.” Several short-term RCTs have demonstrated that curcumin supplementation significantly reduces circulating levels of multiple pro-inflammatory and angiogenic mediators in individuals with obesity. In a randomized controlled study involving 37 obese patients with a 30-day intervention period, Shiva Ganjali et al. reported that daily supplementation with 1,000 mg of curcumin significantly reduced serum levels of interleukin-1 beta (IL-1β), interleukin-4 (IL-4), and vascular endothelial growth factor (VEGF) (20). In addition, a meta-analysis reported significant reductions in IL-1β, IL-4, and VEGF (39). Collectively, these findings provide partial clinical validation of the anti-inflammatory mechanisms proposed by preclinical studies, suggesting that modulation of inflammatory responses may represent a key entry point for curcumin-based interventions in obesity. Nevertheless, most available studies are short-term and involve relatively small sample sizes, which limits the robustness and generalizability of the current evidence base.

Regarding oxidative stress and metabolic homeostasis, clinical evidence likewise aligns with bibliometric hotspots such as “oxidative stress” and “insulin resistance.” A randomized controlled trial conducted by Sahebkar et al. demonstrated that daily supplementation with 1,000 mg of curcumin for 30 days in obese patients significantly reduced serum pro-oxidant-antioxidant balance (PAB) (19). Furthermore, a study by Cicero et al. involving 80 overweight or obese individuals found that a formulation containing 800 mg of phytosomal curcumin improved fasting insulin levels, homeostasis model assessment of insulin resistance (HOMA-IR), triglyceride levels, and selected liver function parameters over a 56-day intervention period (23). These studies support, at the clinical level, the hypothesis that curcumin may influence metabolic status by alleviating oxidative stress and improving insulin sensitivity. Notably, these beneficial effects appear to be more consistently observed in mechanism-based biomarkers than in gross anthropometric outcomes, further underscoring the partial but not yet complete translation of preclinical evidence into clinically measurable anti-obesity effects.

In contrast, evidence regarding traditional obesity outcomes such as body weight, body mass index (BMI), and body fat remains less consistent, revealing a degree of discordance with bibliometric research hotspots related to “weight management.” A meta-analysis reported that short-term supplementation with curcumin at doses of 500–1,000 mg/day significantly reduced body weight, BMI, waist circumference, hip circumference, and body fat percentage (39). However, findings from individual RCTs are not entirely consistent. In a randomized crossover trial, Akram Mohammadi et al. observed that curcumin supplementation (1,000 mg/day) significantly reduced serum triglyceride levels, whereas no significant changes were detected in anthropometric measures such as body weight, BMI, waist circumference, hip circumference, or body fat (18). These discrepancies suggest that the direct effects of curcumin on obesity phenotypes may be modest and may also depend on intervention duration, formulation characteristics, and differences in study populations.

These inconsistent findings regarding body weight and BMI may be attributed to several factors. First, bioavailability remains a major determinant. Standard curcumin formulations exhibit very low oral bioavailability owing to poor aqueous solubility, rapid hepatic metabolism, and extensive intestinal glucuronidation (40, 41). As a result, systemic concentrations achieved by conventional curcumin preparations may be insufficient to induce measurable reductions in body weight or fat mass, even when local anti-inflammatory or metabolic effects are detectable. This dose–response disconnect may partly explain why inflammation-related biomarkers, such as cytokines and PAB, often improve significantly, whereas body weight—a more complex and multifactorial clinical endpoint—remains unchanged, particularly in short-term studies (18–20). In addition to intrinsic bioavailability limitations, formulation heterogeneity may substantially influence clinical outcomes. Trials using bioenhanced formulations, such as curcumin combined with piperine or phytosomal carriers, may achieve higher systemic exposure than studies using conventional curcumin preparations. For example, Cicero et al. (23) used a phytosomal curcumin formulation and observed improvements in insulin resistance and liver-related parameters over a 56-day intervention period, suggesting that enhanced absorption may improve the detectability of metabolically relevant effects. However, large-scale clinical validation of emerging delivery systems, including nanoparticle-encapsulated curcumin and water-dispersible turmeric extracts, remains limited (33). Intervention duration and population heterogeneity may also contribute to inconsistent anthropometric findings. Inflammatory and oxidative stress markers may respond within relatively short intervention periods, whereas meaningful changes in body weight or fat distribution generally require longer follow-up and are likely to be influenced by diet, physical activity, baseline metabolic status, and obesity-related comorbidities.

Taken together, existing clinical studies provide preliminary but incomplete support for bibliometrically identified core research themes, particularly in the domains of inflammation, oxidative stress, and selected metabolic abnormalities. In contrast, the consistency of evidence remains insufficient for endpoints related to body weight and adiposity. This pattern closely mirrors the translational bottleneck identified in the present analysis, namely, the coexistence of abundant mechanistic and preclinical evidence with comparatively limited and heterogeneous clinical findings. Therefore, the inconsistency of current evidence regarding body weight and adiposity should not be interpreted simply as an absence of biological efficacy. Rather, it indicates that the clinical effects of curcumin may be more readily captured by mechanism-related inflammatory and metabolic biomarkers than by direct anthropometric measures. Although curcumin-based anti-obesity research has entered the stage of clinical exploration, the evidentiary chain linking mechanistic insights to clearly defined clinical benefits remains incomplete. Future studies should therefore prioritize adequately powered and longer-term randomized controlled trials using standardized, bioavailable formulations and clearly defined dosing regimens. Future clinical studies may also benefit from more precise patient characterization, stratified analyses based on baseline metabolic and inflammatory status, and improved attention to dose–response relationships, pharmacokinetic characteristics, long-term safety, and concomitant lifestyle or pharmacological interventions.

### 4.5 Research limitations

Several limitations of this study should be acknowledged. First, although both WoSCC and Scopus were searched, WoSCC was used as the primary analytical dataset, whereas Scopus served mainly as an external validation source for publication trends and keyword thematic structures. This strategy improved cross-database robustness, but the lack of a fully integrated multi-database analysis may still have limited dataset comprehensiveness and introduced some degree of selection bias. Second, the analysis was restricted to English-language publications and to articles and reviews, which may have excluded relevant evidence published in other languages or in other document types. Third, differences in database coverage, indexing practices, and update cycles may have affected record retrieval and keyword representation. Another limitation lies in the literature retrieval strategy, which relied on predefined keywords related to curcumin and obesity. Despite the careful construction of the search terms, variations in terminology, indexing practices, and author-selected keywords are unavoidable. Consequently, studies focusing on obesity-related metabolic phenotypes or indirect outcomes may not have been comprehensively captured.

Bibliometric indicators such as publication counts, citation frequency, H-index values, and network centrality primarily reflect academic influence and structural relationships rather than methodological rigor or clinical robustness. Highly cited publications do not necessarily correspond to the highest level of clinical evidence, as citation patterns may also be influenced by publication age, journal visibility, and prevailing research trends. In addition, co-occurrence, clustering, and burst detection analyses are dependent on algorithmic models and parameter settings. Although widely accepted software tools and standardized analytical procedures were employed to enhance reproducibility, a certain degree of subjectivity in parameter selection is inevitable, which may affect cluster resolution and temporal interpretations. Finally, as a bibliometric study, this work provides a macroscopic overview of knowledge structures and research evolution, but cannot replace a critical appraisal of experimental design, mechanistic validity, preclinical reproducibility, or the quality of clinical trials. Therefore, the findings should be interpreted in conjunction with detailed experimental and clinical evidence and should not be considered equivalent to a systematic assessment of efficacy or evidence strength.

### 4.6 Future research directions and key priorities

The bibliometric patterns identified in this study suggest that future research on curcumin and obesity is likely to benefit from deeper integration across mechanistic, technological, and clinical dimensions. Although inflammation-and metabolism-related pathways continue to form the foundation of this field, growing evidence indicates that isolated pathway analyses are insufficient to explain the complex and context-dependent effects of curcumin. Greater emphasis on systems-level approaches, incorporating interactions among gut microbiota, immune regulation, adipose tissue function, and metabolic networks, may provide a more comprehensive understanding of its biological activity and help clarify why mechanistic effects do not always translate into consistent clinical outcomes.

Persistent attention to formulation-related challenges also appears essential. Limited bioavailability remains a major factor restricting clinical consistency, and recent trends highlight increasing interest in nano-delivery systems, composite formulations, and multi-component synergistic strategies. Future studies would benefit from moving beyond proof-of-concept experiments toward mechanistic clarification and standardization, allowing more reliable comparison across studies and facilitating translational reproducibility. In particular, head-to-head comparisons among conventional curcumin, phytosomal formulations, piperine-enhanced preparations, and other bioavailability-optimized systems may help determine which delivery strategies are most suitable for obesity-related clinical applications.

At the clinical level, the existing evidence base underscores the need for more rigorous trial designs. Future randomized controlled trials would be strengthened by larger sample sizes, longer follow-up durations, and multicenter collaboration. Beyond conventional outcomes such as body weight and metabolic indices, incorporation of mechanism-related endpoints—including adipose tissue function and validated biological markers—may help bridge the current gap between mechanistic research and clinical efficacy evaluation. Particular attention should be paid to populations with obesity-associated metabolic dysregulation, such as individuals with metabolic syndrome, type 2 diabetes, and non-alcoholic fatty liver disease, in whom curcumin may have greater potential as an adjunctive intervention. In addition, future studies should evaluate dose–response relationships, long-term safety, and possible interactions with lifestyle interventions or existing pharmacotherapies.

Taken together, these directions reflect a clear transition of the field from predominantly exploratory research toward structured evaluation of clinical value and application optimization. Continued interdisciplinary collaboration and methodologically rigorous research will be crucial for advancing curcumin from a biologically active natural compound to a precisely positioned nutritional intervention with defined clinical relevance in obesity management. Such progress, together with standardized formulations and deeper mechanistic verification, will also be essential for bridging the current translational gap between robust preclinical evidence and heterogeneous clinical findings.

## 5 Conclusion

This bibliometric analysis provides a comprehensive overview of the research landscape, knowledge structure, and evolutionary trends in studies investigating curcumin and obesity. The findings indicate that this field has expanded steadily over the past two decades, with research topics converging on adipose tissue regulation, inflammation, and oxidative stress, metabolic comorbidities, and formulation optimization. Mechanistic evidence consistently supports the role of curcumin as a multi-target modulator acting across interconnected metabolic and inflammatory pathways, while emerging themes such as gut microbiota regulation and advanced delivery systems highlight ongoing paradigm shifts. Despite these advances, gaps remain between mechanistic discoveries and robust clinical validation, underscoring the need for standardized formulations and well-designed clinical studies. The results further suggest that phenotype-oriented research and interdisciplinary integration may enhance translational relevance. Overall, this study clarifies the current status and developmental trajectory of curcumin-related obesity research and provides a structured reference for future mechanistic exploration, clinical evaluation, and application optimization. At the same time, the current evidence suggests that curcumin may be more realistically positioned as a mechanism-targeted adjunctive intervention for obesity-related metabolic dysregulation than as a stand-alone weight-loss agent. Further progress in this field will depend on whether mechanistic insights can be translated into reproducible and clinically meaningful benefits through rigorous and standardized research designs.

## Data Availability

The original contributions presented in the study are included in the article/Supplementary material, further inquiries can be directed to the corresponding author/s.
