# Supplementary figures and images for: A bibliometric analysis of research on the anti-obesity effects of curcumin from 2006 to 2025: knowledge structure, research hotspots, and evolution of frontiers
Source: Front Nutr. 2026 May 28;13:1825692. doi: 10.3389/fnut.2026.1825692 (PMC13253952; doi:10.3389/fnut.2026.1825692)

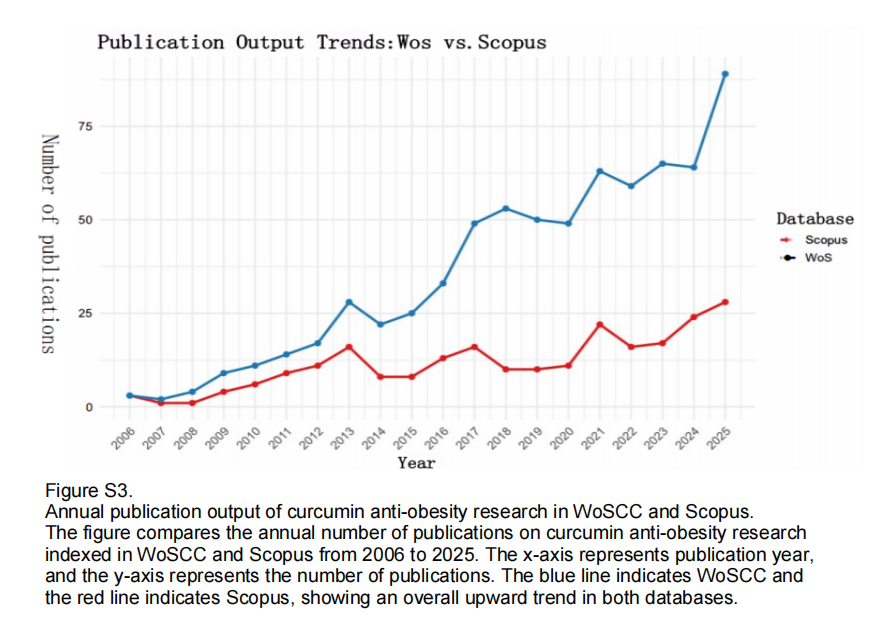

Supplement: Supplementary file 2 [file Image_1.png]

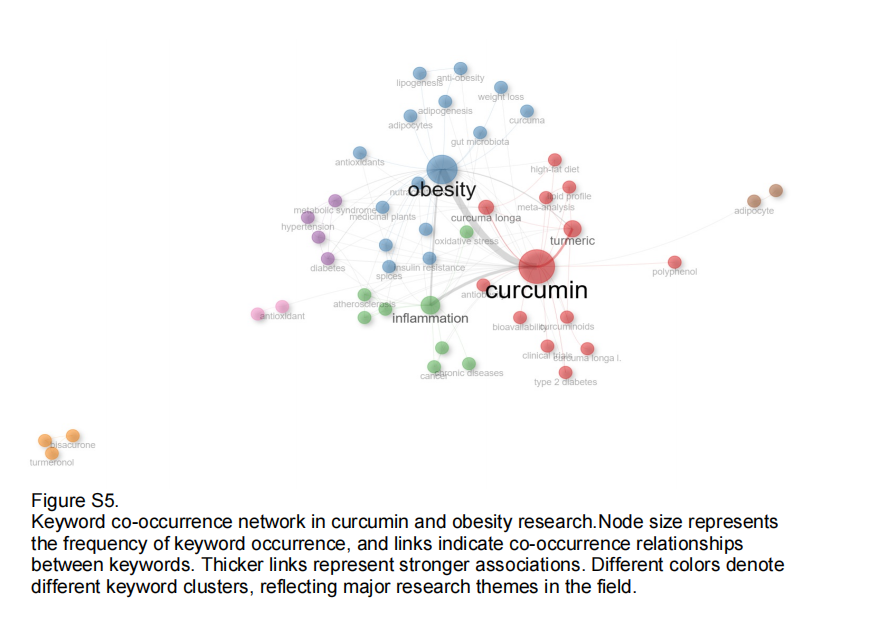

Supplement: Supplementary file 3 [file Image_2.png]
